# Supplementary material for: Should Carbohydrate Intake Be More Liberal during Oral and Enteral Nutrition in Type 2 Diabetic Patients?
Source: Nutrients. 2023 Jan 14;15(2):439. doi: 10.3390/nu15020439 (PMC9863670; doi:10.3390/nu15020439)
Supplement: Supplementary file 1 [file nutrients-15-00439-s001.zip › nutrients-2119544-supplementary.pdf]

Supplementary Table S1. Characteristic of patients included in this study

Patient		Age [years]	Height [cm]	Weight [kg]	Purpose of hospital stay[1]	Comorbidities[2]	Diabetes treatment[3]
**T2DM patients**							
1	M	74	171	111	R, WH	CLI, CHD, COPD	Ins
2	M	57	174	95	R, WH	CLI, CHD, HBP	Ins
3	F	75	150	60	R, WH	CLI, CHD, HBP	Met
4	F	66	165	113	R, WH	CLI, CHD, HBP	Met
5	F	79	154	56	R, WH	CLI	Met
6	F	82	153	60	R	BF	Met
7	M	57	168	94	R, WH	FG, LC	-
8	M	59	180	90	R, WH	CLI, CHD, HBP	Ins, GLP
9	F	70	159	102	R, WH	CLI, VLU	-
10	F	76	158	113	R	VLU	Ins
11	M	70	171	82	R, WH	VLU, CHD, HBP	Ins
12	F	79	170	72	R	CI	-
13	M	89	167	59	R, WH	CLI, CHD, HBP	Sul
14	F	85	165	94	R, WH	VLU, HBP	Ins, Sul
Mean ± STD		72.7 ± 9.8	164.6 ± 8.4	85.8 ± 20.3			
**Non-diabetic patients**							
1	F	76	168	70	R	BF	-
2	M	81	152	76	R, WH	PU	-
3	F	83	160	77	R, WH	VLU	-
4	F	88	162	61	R, WH	VLU	-
5	F	87	170	68	R, WH	VLU	-
6	F	71	170	75	R, WH	CLI, CHD, HBP	-
7	F	66	163	81	R, WH	VLU	-
Mean ± STD		78.9 ± 7.6	163.6 ± 6.0	72.6 ± 6.2			

[1] R – rehabilitation, WH – wound healing

[2] CLI – chronic limb ischemia, CHD – coronary heart disease, COPD- chronic obstructive pulmonary disease, HBP – high blood pressure, BF – bone fractures, FG - Fournier gangrene and abdominal wall necrosis, LC – liver cirrhosis, VLU – venous leg ulcer, CI - cerebral infarction, PU – pressure ulcer

[3] Ins – insulin, Met – metformin, Sul – sulfonylurea, GLP - glucagon-like peptide-1 analogues
